# Highlights in Pathophysiology of the Musculoskeletal System

**DOI:** 10.3390/ijms24076412

**Published:** 2023-03-29

**Authors:** Rosario Barone, Marta A. Szychlinska

**Affiliations:** Department of Biomedicine, Neuroscience and Advanced Diagnostics, University of Palermo, 90127 Palermo, Italy; rosario.barone@unipa.it

The intention of the present Special Issue is to focus on the latest research in the musculoskeletal system, with an emphasis on the molecular mechanisms underlying its pathophysiology, as well as innovative diagnostic tools and therapeutic perspectives. 

The musculoskeletal system is the biggest human organ system, comprising bones, providing support, articular cartilage, allowing joints to slide, ligaments and tendons, connecting all the bones together and muscles, with associated nerves and blood vessels, permitting them to contract and allowing the movement of the human body, which, in turn, underlies the basis of life. 

The mechanobiology of the musculoskeletal system is based on the strong interconnection between the mechanical forces and the biological cues of molecules, cells, and tissues which are connected in the kinetic chain in which one site alterations affect all the tissues and organs. It means that, when the tendon or ligament is damaged, the released metabolites, cytokines, extracellular vesicles, and other mediators affect the cartilage and bone locally, as well as the surrounding muscles, and even distant tissues due to the instauration of a systemic inflammatory response triggering a chain reaction throughout the body. The management of the pathological processes established at the molecular level requires a multidisciplinary approach based on a holistic view of the entire system in terms of prevention, early diagnosis, and innovative therapeutic approaches. 

The holistic view of the musculoskeletal system as an interdependent organ system has been highlighted by the authors contributing to the present special issue. For instance, Eldjoudi and co-authors [1], in their interesting review article, summarize the most recent findings concerning the involvement of an adipokine, namely, leptin, in the cross-talking networks involved in the pathogenesis of osteoarthritis (OA) and rheumatoid arthritis (RA). The authors highlight the ability of leptin to promote cartilage catabolism through systemic and local inflammatory responses, underlying the pathophysiological interdependence between the organs and tissues. The vicious circle between the lipid metabolism, inflammatory cytokines, and skeletal muscle has been highlighted in a review by Mangano et al. [2], in which the authors discuss this interdependent relationship in referment to cachexia [3]. The latter is a multifactorial and multi-organ syndrome, representing a major cause of morbidity and mortality in late-stage chronic diseases, which requires a multidisciplinary therapeutic approach. In particular, the authors discuss a beneficial effect of physical activity in interrupting the cited above vicious circle, suggesting that the molecular basis underlying the application of mechanical forces could provide important insights on the interactions between organs and the systemic mediators involved in body homeostasis, as supported by other studies [4,5,6,7]. Another review, underlying this important interconnection between different organs and systems, is a contribution of Cheng et al. [8]. The authors examine the muscle wasting physiopathology, based on the muscle protein metabolism dysregulation and impaired muscle cell regeneration, in patients with chronic kidney disease (CKD). In particular, the authors focus on the involvement of myokines signaling pathways, and on the microRNA- and gut microbiota-mediated regulation of muscle mass and myogenesis, highlighting the importance of considering the inter-systemic connection in managing the muscle wasting condition. Myokines are molecules released during muscle contraction, reported to play a positive or negative role in muscle function and metabolism homeostasis [9]. In the study by Zerlotin et al. [10], the authors hypostasized the potential mechanism underlying the high serum levels of irisin, a myokine which has a positive effect on muscle mass, in patients suffering from dermatomyositis and immune-mediated necrotizing myopathy, two rare diseases belonging to the group of idiopathic inflammatory myopathies characterized by muscle atrophy. Surprisingly, the authors found decreased levels of FNDC5, the precursor of irisin, and high levels of ADAM10, a metalloproteinase responsible for FNDC5 cleavage, in the muscle biopsies of the patients, suggesting that the overexpression of ADAM10 might be directly implied in the FNDC5/irisin unbalance.

The muscle atrophy, fibrosis, and inflammation are the main causes of muscle weakness but also of the surrounding tissue impairment [11]. There are several factors contributing to this pathologic condition. Among others, the study by Caceres-Ayala et al. [12] explores the effects of binge alcohol drinking in the development of alcoholic myopathy in rats. The authors reported that the rats subjected to episodic alcohol consumption presented muscle atrophy, evidenced by reduced fiber size and the increased expression of atrophic genes. Moreover, increased fibrotic, oxidative stress, and inflammation markers were reported, suggesting that episodic binge-like ethanol exposure alters contractile capacity and increases fatigue, affecting the general muscle performance. Muscle atrophy also represents one of the most important symptoms of congenital genetic muscle disorders, such as centronuclear myopathy, caused by mutations in the genes encoding proteins involved in membrane trafficking and remodeling. In an interesting study by Bayones et al. [13], the authors explore the mutations of dynamin-2 (namely, p.A618T and p.S619L), a mechano-GTPase that remodels membrane and actin filaments, and their influence on myoblast exocytosis. The results of the study indicate that both mutations impair the exocytosis by affecting the fusion pore dynamics and disrupting the formation of actin filaments, which may disturb the plasmalemma expression of functional proteins such as the glucose transporter GLUT4 in skeletal muscle cells, impacting the physiology of the skeletal muscle tissue. Another rare genetic disease leading to progressive muscle wasting is represented by Duchenne muscular dystrophy (DMD). In a study by Giovarelli et al. [14], a characterization of progressive skeletal muscle fibrosis in the mouse model of DMD has been investigated. The authors reported that fibrosis mostly affects diaphragm and quadriceps with a higher collagen cross-linking and the inhibition of metalloproteinases that contribute differently to progressive collagen accumulation during fibrotic remodeling, providing new insights for new therapeutic targets for the disease [15]. Furthermore, Merckx et al. [16] investigated osmoregulatory changes in the mdx mouse model by examining the expression of osmolyte pathway members and highlighting their potential as a research interest and future therapeutic target in dystrophinopathy. Furthermore, in the in vitro study on dexamethasone-induced damaged C2C12-derived myotubes by Micheli et al. [17], the authors investigated the protective potential of exogenous (glucoraphanin, L-cysteine, and 3-mercaptopyruvate) and endogenous sources of H2S, an endogenous gasotransmitter involved in skeletal muscle wasting and homeostasis. The authors concluded that glucoraphanin, a natural H2S-releasing molecule, appears effective for preventing skeletal muscle damage, suggesting that its supplementation could be useful in the management of sarcopenia. 

Aging represents another leading risk factor for musculoskeletal disorders, considering also the metabolic alterations that occur with aging, that may lead to undernutrition, dysbiosis, and other digestive system-related disorders, which, in turn, can worsen age-related variations, as in a vicious circle [18,19]. The impact of undernutrition on aged skeletal muscle has been explored by Barbé et al. [20] through the skeletal muscle proteome analysis in old rats. The results revealed that undernutrition has a profound effect on muscle proteome in a specific muscle-type manner. In particular, it was shown that slow-twitch muscles showed a differential expression in the proteins implied in the energy metabolism, whereas fast-twitch muscle mainly showed changes in protein turnover. 

Furthermore, skeletal muscle is considered the main site for insulin-stimulated glucose uptake and therefore, a primary target for insulin resistance in the human body. In a study by Grunwald et al. [21], the localization and expression of the proteins related to GLUT4-mediated glucose uptake via AMPKα or AKT in human skeletal muscle tissue from patients with statin intake has been investigated. The authors concluded that statin treatment activates the AMPKα-dependent glucose uptake and remains active after long-term statin treatment, suggesting that the permanent blocking of its insulin-dependent counterpart AKT activation may lead to metabolic inflexibility and insulin resistance in the long run, and this may be a direct consequence of statin treatment.

Recently, in the perspective of the management of articular cartilage degenerative disorders, a multidisciplinary approach based on the construction of “smart” biomaterials for tissue engineering, able to interact both with the damaged and surrounding tissues, raised increasing attention among the scientific community [22,23,24,25]. In the review by Rizzo et al. [26], the authors highlight the potential of the growth factor mimetic peptides in an osteochondral tissue engineering approach, discussing their ability to act as active molecules and enrichment motifs for the functionalization of biomaterials and exhibiting chondrogenic/osteogenic differentiation properties. Moreover, the authors report the current knowledge of the use of phage display peptides as a promising tool in osteochondral repair strategies. Furthermore, the involvement of sirtuins, enzymes involved in the cell metabolism and the regulation of several cellular functions, has been discussed in relation to the pathophysiology of RA by Pniewierska-Baran et al. [27]. The authors suggest that the activation of these small molecules is able to limit the development of autoimmune and inflammatory diseases such as RA by promoting cell survival pathways and attenuating the inflammatory process. The pathophysiological mechanisms of RA have been also explored by Gyebrovszki et al. [28] in a study focusing on the role of the anti-citrullinated peptide antibodies (ACPA)-IgG region N-glycosylation. The results demonstrate that both the sialylation and galactosylation levels of ACPA-IgG negatively correlate with inflammation-related clinical parameters in RA and that ACPA-IgG-induced inflammation inversely correlates with the relative intensities of the G0 form of the IgG Fc glycan, suggesting its implication in RA physiopathology [29].

Finally, we wish to highlight the contributions to the present SI, focusing on the innovations in the examination and diagnostic tools used in the musculoskeletal disorder evaluation and treatment. For instance, the study by Kamp et al. [30] evaluated and reported on the apparent tissue sodium content (aTSC) and sodium relaxation times for the Achilles tendons (ATs) by using sodium magnetic resonance imaging (MRI) to serve as a better comparable parameter between different studies regarding the examinations of diseased ATs with sodium MRI. Still, based on in silico, in situ, and in vivo studies, the study by Radke et al. [31] developed and reported a new method for the quantitative chemical exchange saturation transfer (qCEST) technique, a promising bio-sensitive MRI technique, in systems with multiple proton pools, to assess the biochemical composition of the intervertebral discs, allowing for the timely diagnosis of degenerative diseases. Moreover, the validity of the dual innervation technique for the facial paralysis treatment has been reported in a study by Watanabe et al. [32]. The results of immunohistochemical and microarray analysis on the masseter muscle of rats subjected to masseteric and hypoglossal nerves suture, reported oxidative characteristics, supporting the validity of the technique. 

We express thanks to all the authors who contributed to this Special Issue, who added, through their researches, a small brick to this extensive wall of knowledge regarding the musculoskeletal disorder physiopathology, diagnosis, and therapeutic perspectives, providing new molecular-level insights and inviting the scientific community to further deepen research into the treated aspects.
